# Local A‐Site Layering in Rare‐Earth Orthochromite Perovskites by Solution Synthesis

**DOI:** 10.1002/chem.201604766

**Published:** 2016-11-03

**Authors:** Luke M. Daniels, Reza J. Kashtiban, Demie Kepaptsoglou, Quentin M. Ramasse, Jeremy Sloan, Richard I. Walton

**Affiliations:** ^1^ Department of ChemistryUniversity of WarwickCoventryCV4 7ALUK; ^2^ Department of PhysicsUniversity of WarwickCoventryCV4 7ALUK; ^3^ SuperSTEM LaboratorySciTech Daresbury CampusDaresburyWA4 4ADUK

**Keywords:** chromites, electron energy loss spectroscopy (EELS), hydrothermal synthesis, magnetism, perovskites

## Abstract

Cation size effects were examined in the mixed A‐site perovskites La_0.5_Sm_0.5_CrO_3_ and La_0.5_Tb_0.5_CrO_3_ prepared through both hydrothermal and solid‐state methods. Atomically resolved electron energy loss spectroscopy (EELS) in the transmission electron microscope shows that while the La and Sm cations are randomly distributed, increased cation‐radius variance in La_0.5_Tb_0.5_CrO_3_ results in regions of localised La and Tb layers, an atomic arrangement exclusive to the hydrothermally prepared material. Solid‐state preparation gives lower homogeneity resulting in separate nanoscale regions rich in La^3+^ and Tb^3+^. The A‐site layering in hydrothermal La_0.5_Tb_0.5_CrO_3_ is randomised upon annealing at high temperature, resulting in magnetic behaviour that is dependent on synthesis route.

Perovskites ABX_3_ are one of the most versatile group of materials in solid‐state chemistry with respect to valence and ionic radii of the possible incorporated cations A and B, as well choice of anion X, from oxide to halides. This compositional and structural flexibility allows a variety of distortions from the archetypical cubic perovskite structure mediated through phenomena such as cation displacements, octahedral tilts, ordered vacancies and Jahn–Teller effects. For perovskite oxides, this gives rise to interesting and functional properties, such as high‐temperature superconductivity, piezoelectricity and ferroelectricity, colossal magnetoresistance, multiferroism and catalytic activity.^1^

More subtle cation order/disorder effects can result when multiple metals share the same crystallographic position;^2^ in particular, ordered cation arrangements are probable when their charge and/or ionic radii differ sufficiently.^3^ Ordered arrangements of mixed B site cations are observed more frequently than those of the A site.^4^ Rock‐salt ordering of B sites in compositions A_2_BB′X_6_ is commonplace, while layered configurations of the A sites in compositions AA′B_2_X_6_ or AA′BB′X_6_ are found when they provide lower bonding strains with the neighbouring anions.^5^ For those materials, layered A site arrangements result from effects driven by the B site ordering, such as anion vacancies, A site vacancies and second‐order Jahn–Teller distortions.^6^ A review of the literature shows that it is rare for stoichiometric perovskites to exhibit long‐range A site order in the absence of an ordered *B*/*B*′ sublattice, with layering effects being observed only on the local scale without B site ordering in materials such as NaLa(*BB*′)O_6_ (*B*=Fe or Mn, and *B*′=Nb or Ta), in which B site second‐order Jahn–Teller effects and charge difference between the Na^+^ and La^3+^ are the driving mechanisms.^7^ Structural order may be driven by anion vacancies, such as the ordered cation arrangements of the triple perovskites, YBa_2_Cu_3_O_7‐*x*_ and YBa_2_Fe_3_O_8+*x*_, with separate eight‐ and ten‐coordinate A sites, respectively.^8^ In this paper we investigate the possibility of A site order in chromite perovskites, in which by using a single B site cation and isovalent lanthanides on the A site we negate the common mechanisms for A site order. In doing so, we are able to observe the direct effects of cation radius variance on A site ordering in mixed rare‐earth orthochromite perovskites.

Rare‐earth orthochromite perovskites are already known to exhibit a plethora of properties,^9^ and it is their magnetoelectric properties that have produced most interest, firstly with the theoretical prediction of a spontaneous electrical polarisation,^10^ followed by the experimental observation of both canted antiferromagnetism and polarisation in several *R*CrO_3_ oxides (*R*=Sm^3+^, Gd^3+^, Tb^3+^, Er^3+^ and Tm^3+^),^11^ and observations of spin–phonon couplings as a result of magnetically induced symmetry breaking.^12^ Recently, it was shown that the presence of a magnetic *R* ^3+^ cation is not necessary to generate such behaviour,^13^ and current theoretical work suggests that these polarisations can be tuned by external magnetic fields.^14^ We have previously shown how hydrothermal conditions at approximately 400 °C can be used to form well‐crystallised samples of rare‐earth orthochromites with magnetic properties that match those from conventional synthesis.^15^ Herein, we compare synthesis routes of new mixed A site examples, not previously reported by any synthetic method, in which extreme A‐site radius variance leads to preparation‐dependent local structure and, in turn, magnetic properties.

Crystalline La_0.5_Sm_0.5_CrO_3_ and La_0.5_Tb_0.5_CrO_3_ were each produced through two distinct synthetic routes involving very different reaction temperatures; the hydrothermal treatment of an amorphous mixed‐metal hydroxide at approximately 400 °C,15a and conventional solid‐state synthesis, in which the same amorphous mixed‐metal precursors were fired at temperatures of 1200 °C or higher in air. Hydrothermal synthesis produced phase‐pure powders of La_0.5_Sm_0.5_CrO_3_ and La_0.5_Tb_0.5_CrO_3_ at temperatures of 375 °C (for 6 h) and 410 °C (for 12 h), respectively, whilst solid‐state reactions were performed at 1200 °C (for 12 h) and 1400 °C (for 96 h).

The resulting product of each reaction (Figure 1 a, b and the Supporting Information, Figure S1) is phase pure by powder X‐ray diffraction (PXRD). The patterns of all four materials were indexed to the orthorhombic space group *Pnma*, and Rietveld analysis showed their structures to be that of the classical GdFeO_3_ distorted perovskite. No evidence for ordering effects of separate lanthanide sites was observed in the powder diffraction data (such as a contracted unit cell that might result from segregation of A sites into layers), and both the La^3+^ and Sm^3+^, and the La^3+^ and Tb^3+^, were modelled on the same crystallographic 4*c* position (*x*, 1/4 , *z*) with split occupancy. The refined lattice parameters of both materials display a linear dependence between the respective single lanthanide end members, following Vegard's law (the Supporting Information, Table S1 and Figure S2). The smaller mean ionic radius of the A site in La_0.5_Tb_0.5_CrO_3_ results in increased tilting of the CrO_6_ octahedra, compared to La_0.5_Sm_0.5_CrO_3_, giving a smaller unit cell volume.

**Figure 1:**
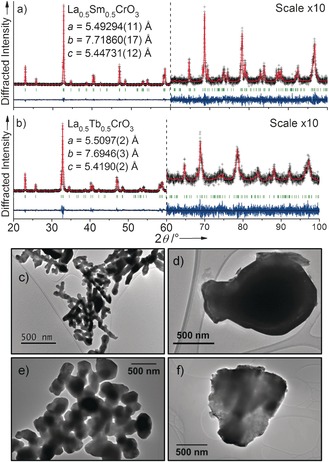
Rietveld refinements performed against room temperature PXRD data (*λ*=1.54056 Å) of hydrothermal samples of a) La_0.5_Sm_0.5_CrO_3_ and b) La_0.5_Tb_0.5_CrO_3_. The regions 60≤2*θ*≤100 are scaled to display the fits at higher angle. Observed data (black crosses), calculated (red line), and difference (blue line) patterns are shown. Green tick marks denote positions of expected reflections for space group *Pnma*. TEM images compare particle morphologies between hydrothermal and solid‐state‐prepared materials of c), d) La_0.5_Sm_0.5_CrO_3_ and e), f) La_0.5_Tb_0.5_CrO_3_, respectively.

The lattice parameters of the materials produced through the two synthesis routes agree within 0.2 % for La_0.5_Sm_0.5_CrO_3_ and 0.4 % for La_0.5_Tb_0.5_CrO_3_ (the Supporting Information, Table S2). PXRD suggests that the hydrothermally prepared materials are the more crystalline powders, as broader peaks were observed for the solid‐state samples. In addition, transmission electron microscopy (TEM) shows that hydrothermal treatment of the amorphous precursors leads to increased homogeneity of particle morphology and size compared to those fired at 1200 °C or above. Dendritic morphologies of a few μm in size are observed for both hydrothermal samples (Figure 1 c, e), whereas a much greater distribution of particle size is observed for the solid‐state prepared materials, ranging from 0.1 up to 10 μm (Figure 1 d, f).

Raman spectra (the Supporting Information, Figure S3) display mode‐softening behaviour towards LaCrO_3_, as was observed in all *R*CrO_3_,15c and the increased breadth of the observed bands in La_0.5_Tb_0.5_CrO_3_ gave an indication of compositional disorder present on the A site. Compositional disorder in *A_x_A*′_1−*x*_ *BX*_3_ materials can be quantified by the statistical variance (*σ* ^2^) of the ionic radii of the two A‐site cations present, with higher variance referring to greater size disparity. The importance of this was emphasised by Attfield, who showed cation‐radius variance to affect the magnetoresistive properties of doped lanthanide manganite materials (La_1−*x*_Sr_*x*_MnO_3_) and cuprate superconductors (La_2‐*x*_Sr_*x*_CuO_4_).^16^ To the best of our knowledge, the new La_0.5_Tb_0.5_CrO_3_ composition has the largest A‐site radius variance of any mixed rare‐earth chromite reported to date, 25 % greater than that of La_0.5_Gd_0.5_CrO_3_,^17^ (Figure 2 a, b). It is possible that the longer synthesis durations and higher temperatures required for La_0.5_Tb_0.5_CrO_3_ by both synthetic routes (the Supporting Information, Figure S4) are associated with this increased radius variance compared to those of La_0.5_Sm_0.5_CrO_3_. Using the lower temperature and shorter reaction time needed for La_0.5_Sm_0.5_CrO_3_ resulted in the presence of hydroxide impurities in the hydrothermal La_0.5_Tb_0.5_CrO_3_ material. Attempts were made to produce solid solutions with greater variance, such as La_0.5_Ho_0.5_CrO_3_ and La_0.5_Yb_0.5_CrO_3_ (the Supporting information, Figure S5); however, these did not result in single phases, suggesting the existence of a synthetic A‐site radius variance limit.

**Figure 2:**
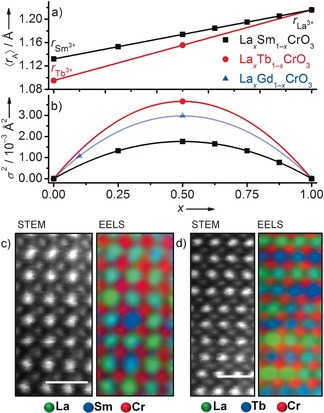
Variation in mean rare‐earth A‐site cation: a) radii and b) variance for La_*x*_Sm_1−*x*_CrO_3_ (black), La_*x*_Tb_1−*x*_CrO_3_ (red) solid solutions. Variance of La_*x*_Gd_1−*x*_CrO_3_ reported previously is included for comparison (blue).^17^ Points along each line represent solid solutions synthesised in the present and previous studies.15a HAADF‐STEM images and corresponding EELS maps of hydrothermal c) La_0.5_Sm_0.5_CrO_3_ and d) La_0.5_Tb_0.5_CrO_3_ crystallites aligned along the [101] zone axis. Scale bars represent 1 nm.

High‐angle annular dark field scanning transmission electron microscopy (HAADF‐STEM) and electron energy loss spectroscopy (EELS) were performed on crystallites aligned along the [101] zone axis in the *Pnma* setting (equivalent to pseudo cubic [100] projection of *Pm* $\bar{3}$ *m*), which provided large separation between neighbouring A and B site columns, ideal for EELS mapping. The lanthanides appeared as brighter columns in the HAADF images (the Supporting Information, Figure S6), and distinguishing between the two rare‐earths in each material is difficult due to their similar values of *Z* (*Z*_La_=57, *Z*_Sm_=62 and *Z*_Tb_=65). Although direct observations of local A‐site cation ordering have previously been made by using HAADF‐STEM,^7^ and local cation chemistry of complex perovskites can in some cases be determined by imaging alone,^18^ the contrast between the lanthanides used in the current study requires the use of EELS for the pairs to be distinguished in practice. The EELS maps (Figure 2 c, d) showed atomic‐scale differences in lanthanide distribution between hydrothermal La_0.5_Sm_0.5_CrO_3_ and La_0.5_Tb_0.5_CrO_3_. Localised layered‐like ordering of the A‐site cations was observed in the La_0.5_Tb_0.5_CrO_3_ sample, Figure 2 d, compared to random distributions present in La_0.5_Sm_0.5_CrO_3_ (Figure 2 c). EELS spectra were collected from numerous crystallites in each sample to ensure that these observations represented the bulk sample (the Supporting Information, Figure S7, S8, S9 and S10). The only plausible explanation for these observations is the increased A‐site radius variance of the La_0.5_Tb_0.5_CrO_3_ composition, because there is no possible influence from vacancy ordering, charge, or Jahn–Teller effects, as has been seen in other known A‐site ordered perovskites. The formation of these La and Tb layers must only result from the size‐induced strain that exists between each environment local to the larger La^3+^ and smaller Tb^3+^; the segregation of the two different sized lanthanides into layers likely reduces this strain within the local structure. The observation of such layering suggests that the structural distortion, typically mediated through octahedral tilting in perovskites, is inhomogeneous. The short scales on which these inhomogeneities exist are below the observable limit of other techniques, such as PXRD and Raman scattering, both of which present no evidence for long range lattice ordering of the A‐site cations, and so a tilt system cannot be assigned.

The two methods used to synthesise these solid solutions also lead to different atomic‐scale variations in lanthanide distribution (Figure 3). For La_0.5_Sm_0.5_CrO_3_, the EELS maps of samples prepared by hydrothermal and solid‐state methods showed a similar, random, lanthanide distribution. However, unlike the relatively homogeneous distributions of La_0.5_Sm_0.5_CrO_3_, the two La_0.5_Tb_0.5_CrO_3_ materials are significantly different from each other, with no evidence of the layered‐like hydrothermal arrangements being present in the solid‐state material. Instead in the solid‐state sample of La_0.5_Tb_0.5_CrO_3_, large domains of La‐rich and Tb‐rich regions, several nanometres in size, were observed, large enough to be observed through careful analysis of the STEM images (the Supporting Information, Figure S11), which showed separate regions of LaCrO_3_ and TbCrO_3_, with different degrees of cation displacement. This suggests the formation of the locally layered hydrothermal material represents a metastable phase. This was supported by differential scanning calorimetry (DSC), which showed a thermal event exclusive to hydrothermal La_0.5_Tb_0.5_CrO_3_ at 1200 °C compared to the solid‐state and annealed as‐made materials (the Supporting Information, Figure S12). This is indicative that the layered cation configurations of hydrothermal La_0.5_Tb_0.5_CrO_3_ are randomised upon annealing to high temperature with the local A‐site ordering being diminished. The hydrothermal synthesis route is already known to produce metastable layered perovskite phases, such as LaBaMn_2_O_6_, with ceramic synthesis leading to the disordered La_0.5_Ba_0.5_MnO_3_,^19^ but in that case, the A‐site cations have different charges and the B‐site is mixed valent. Indeed, in other mixed lanthanide–barium manganites, the choice of synthesis routes may result in either ordered or disordered A site distributions.^20^ Previously, thermal destabilisation of A site cationic order at 1100 K has been reported in YBaMn_2_O_6_,^21^ resulting in the random A‐site distributions of Y_0.5_Ba_0.5_MnO_3_.

**Figure 3:**
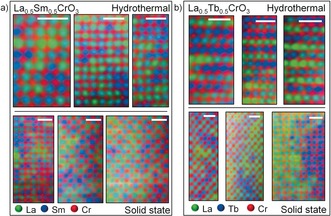
Comparison of EELS maps recorded along the [101] direction of hydrothermal (upper) and solid state (lower) materials in a) La_0.5_Sm_0.5_CrO_3_ and b) La_0.5_Tb_0.5_CrO_3_. The white scale bars in each image represent 1 nm.

The local ordering of A‐site metals manifests itself in bulk magnetic properties. We note that the temperature dependence of the magnetic susceptibility of hydrothermal La_0.5_Tb_0.5_CrO_3_ (the Supporting Information, Figure S13) is indicative of a homogeneous solid solution, exhibiting a linear trend in *T*_N_ (arising from ordering of Cr^3+^ spins above 200 K) between the end members. The decrease in *T*_N_ towards TbCrO_3_ highlights the increased distortion of the structure and is greatly dependent on virtual charge transfer between the Cr^3+^ t_2g_ and e_g_ orbitals, which are hybridised as a result of π and σ bond overlap.^22^ The small ferromagnetic component induced by the canted Cr^3+^ AFM sublattice produced a polarisation of the paramagnetic Tb^3+^ spins, which decouple only at low temperatures to order in an AFM fashion.^23^ This is represented by the observed downturn in the data at low temperatures.

Clear differences were observed in the susceptibility data of La_0.5_Tb_0.5_CrO_3_ produced through hydrothermal and solid‐state techniques (Figure 4 a), which must be a consequence of the atomic‐scale lanthanide distributions described above. The lower *T*_N_ observed for hydrothermal La_0.5_Tb_0.5_CrO_3_ compared to the solid‐state and annealed materials (Figure 4 b) must result from the localised A‐site ordering, with the more distorted Tb^3+^‐rich layers disrupting the superexchange interactions between adjacent Cr^3+^ spins. In contrast, minor differences were observed between the magnetic susceptibility data of La_0.5_Sm_0.5_CrO_3_ materials prepared by different methods (Figure 4 c), with all samples displaying similar low‐temperature behaviour, and which is consistent with previously obtained data from the same composition, in which spin reorientation of chromium gave a broad ordering feature.15a

**Figure 4:**
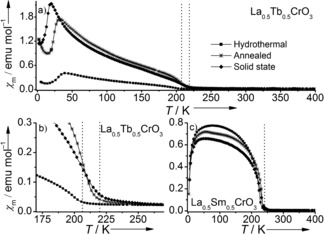
Temperature dependence of molar magnetic susceptibility data (FCC) for a) La_0.5_Tb_0.5_CrO_3_ prepared through hydrothermal (black line), annealed (red line) and solid‐state (blue line) synthetic methods in an applied field of 100 Oe. An enlarged plot of the *T*_N_ region is shown in b), while c) shows the same FCC data for La_0.5_Sm_0.5_CrO_3_. The red dotted lines indicate ordering of the Cr^3+^ spins at *T*_N_.

In La_0.5_Tb_0.5_CrO_3_, further differences in magnetic behaviour between the hydrothermal and solid‐state materials arise at low temperatures, when the hydrothermal sample exhibited a higher Tb^3+^ ordering temperature (ca. 25 K) and much lower susceptibility. We propose that the exchange pathways for Tb‐O‐Tb interactions form across the edge‐to‐edge diagonal of the primitive ABO_3_ cell, giving rise to two‐dimensional interactions occurring within the layered arrangement, and are not able to form a 3D network (Figure 5 a). Interactions between each Tb^3+^ layer are inhibited by the intercalating layers of nonmagnetic La^3+^ (Figure 5 c). For the solid‐state material, the reduced Tb^3+^ ordering temperature and increased susceptibility observed below *T*_N_ resulted from the 3D exchange pathways that can form within the nanoscale regions of TbCrO_3_ (Figure 5 b), which dominate the susceptibility of La_0.5_Tb_0.5_CrO_3_ at low temperature.

**Figure 5:**
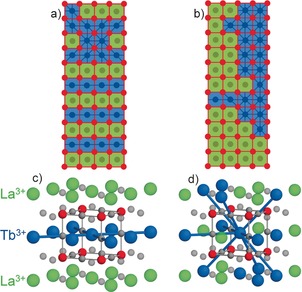
Schematic structural diagrams of La_0.5_Tb_0.5_CrO_3_ perovskite viewed along the [101] direction showing how lanthanide distributions influence the formation of magnetic superexchange pathways between neighbouring Tb^3+^ cations. Distributions a) and b) are constructed from actual EELS maps shown in Figure 3 b. Each square represents a single *AB*O_3_ unit, the colour detailing the lanthanide present at its centre (green La^3+^, blue Tb^3+^). The B‐site lattice is shown in red and red lines show AFM superexchange pathways between neighbouring Cr^3+^ ions, which are affected only by structural distortion. Blue lines represent magnetic‐exchange pathways between Tb^3+^ cations, in which they can form. The environments local to a Tb^3+^ cation are shown in c) the layered hydrothermal structure, and d) with random lanthanide distribution. Atom colours: La green, Tb blue, Cr red, and O grey. Tb‐O‐Tb superexchange pathways are shown as blue lines.

Changes in the magnetic susceptibility were observed when annealing the as‐made hydrothermal La_0.5_Tb_0.5_CrO_3_ to 1400 °C, supporting the DSC measurements that indicated a randomisation of the layered hydrothermal configuration. Annealing to 1400 °C showed a maximum susceptibility comparable to solid‐state La_0.5_Tb_0.5_CrO_3_ but a low‐temperature line shape similar to that of the hydrothermal method. The DSC data suggested that randomisation occurred at 1000 °C, but required the high temperature of 1400 °C to achieve completion. The more randomised distribution of lanthanides would allow increased super‐exchange interactions compared to the layered arrangement (Figure 5 d), giving rise to a much larger magnetic response below *T*_N_.

In conclusion, we have presented evidence of local A‐site layering in a perovskite structure induced exclusively by A‐site cation size variance. This order is then removed by high‐temperature annealing, giving randomised distributions of the A‐site substituents. The locally ordered A site cation distribution in hydrothermal La_0.5_Tb_0.5_CrO_3_ arises only from the size difference between the A‐site metals: their radius variance appears to be at the limit of what is synthetically possible, as was evidenced by the solid‐state sample, which shows significant nanoscale separation of the lanthanides. Other literature examples of A‐site ordering in perovskites are associated with B‐site ordering, and/or charge difference between A ‐site metals, or oxide vacancies. Locally ordered hydrothermal La_0.5_Tb_0.5_CrO_3_ is thus a unique metastable phase with distinct low‐temperature magnetic behaviour, inaccessible through conventional ceramic synthesis. Assuming the metals are homogeneously distributed in solution during synthesis, we speculate that this gives the structure the kinetic possibility of ordering at the point of crystal growth, something not possible by solid‐state reactions, which are limited by the much slower diffusion of ions in the solid state.

## Experimental Section

The synthesis of La_*x*_Sm_1−*x*_CrO_3_ (*x*=0.5) was performed by using the high‐temperature hydrothermal treatment of an amorphous mixed‐metal hydroxide precursor detailed previously.15a The La_*x*_Tb_1−*x*_CrO_3_ (*x*=0.5) amorphous precursor was produced by the same method; however, pure samples of the perovskite were formed only after 12 h of hydrothermal treatment at 410 °C, under 200+ bar of autogeneous pressure, compared to the shorter 6 h reactions at 375 °C for La_0.5_Sm_0.5_CrO_3_. For the conventional solid‐state syntheses, the same amorphous precursors were used for these reactions. The powdered precursors were placed in alumina crucibles and fired at 1200 °C for 12 h for La_0.5_Sm_0.5_CrO_3_, whereas the synthesis of La_0.5_Tb_0.5_CrO_3_ involved a total of four 24 h firing cycles to 1400 °C, with regrinding of the powder in between each cycle (the Supporting Information, Figure S14).

High‐resolution powder X‐ray diffraction (PXRD) data were collected by using a Panalytical X′Pert Pro MPD (Cu_Kα1_, *λ*=1.54056 Å). Rietveld refinements against the data were performed by using TOPAS‐Academic implemented with jEdit (Versions 4.1 and 4.3.1, respectively).^24^ The magnetic properties of the solid solutions were investigated by using a quantum design magnetic property measurement system (MPMS) SQUID magnetometer. The field‐cooled cooling (FCC) data were collected in an applied magnetic field of 100 Oe. For STEM imaging and EELS, polycrystalline samples were ultrasonically dispersed in a methanol suspension onto lacy carbon film reinforced by a copper grid. TEM images were acquired using a JEOL 2100 instrument equipped with a LaB_6_ filament operating at 200 kV, while HRTEM images were acquired using a third‐order (*C*_3_) aberration corrected JEOL ARM200F operating at a voltage of 200 kV. High‐angle annular dark field (HAADF) scanning transmission electron microscopy (STEM) imaging was performed by using a Nion Ultrastem 100 microscope with cold‐field emission gun operating at 100 kV, equipped with a Gatan Enfina spectrometer at the SuperSTEM facility in Daresbury, U.K. The probe‐forming optics were configured to form an approximately 0.9 Å probe (full width at half‐maximum) with a convergence angle of 30 mrad and a probe current of 100 pA. The native energy spread of the electron probe was 0.35 eV, and the collection semi‐angle for the EELS measurements was 36 mrad. Chemical maps were produced by rastering the electron probe serially across a defined region and collecting an EEL spectrum at each point. Chemical maps were created by integrating at each point of these spectrum images the spectrum intensity over an approximately 60 eV window above the Cr L_2,3_, La M_4,5_, Tb M_4,5_ and Sm M_4,5_ EELS edge onsets after background subtraction by using a linear combination of power laws using the ImageJ image processing software implemented with the Cornell spectrum imaging (CSI) plugin.^25^ The individual EELS maps for Cr, La, Sm and Tb were denoised through smoothing in ImageJ before combining into single RGB plots.

## Supporting information

As a service to our authors and readers, this journal provides supporting information supplied by the authors. Such materials are peer reviewed and may be re‐organized for online delivery, but are not copy‐edited or typeset. Technical support issues arising from supporting information (other than missing files) should be addressed to the authors.

Supplementary
