# Supplementary material for: Local A‐Site Layering in Rare‐Earth Orthochromite Perovskites by Solution Synthesis
Source: Chemistry. 2016 Nov 3;22(51):18362–7. doi: 10.1002/chem.201604766 (PMC5216904; doi:10.1002/chem.201604766)
Supplement: Supplementary file 1 — Supplementary [file CHEM-22-18362-s001.pdf]

CHEMISTRY
A European Journal

Supporting Information

Local A-Site Layering in Rare-Earth Orthochromite Perovskites by Solution Synthesis

Luke M. Daniels,[a] Reza J. Kashtiban,[b] Demie Kepaptsoglou,[c] Quentin M. Ramasse,[c] Jeremy Sloan,[b] and Richard I. Walton*[a]

chem_201604766_sm_miscellaneous_information.pdf

Supporting Information

for

Local A-Site Ordering in Rare-Earth Orthochromite Perovskites via Solution Synthesis

*Luke M. Daniels, Reza J. Kashtiban, Despoina-Maria Kepaptsoglou, Quentin M. Ramasse, Jeremy Sloan and Richard I. Walton**

Contents

S1 Further Synthesis Details	2
S2 Further Characterisation Details	2
S3 Crystallographic Data	3
S4 Raman Scattering	5
S5 Synthesis of $La_{0.5}Tb_{0.5}CrO_3$	6
S6 Attempted syntheses of other mixed rare-earth chromites	6
S7 HAADF-STEM & EELS	7
S8 Differential Scanning Calorimetry (DSC)	13
S9 Magnetic susceptibility.	14
S10 Annealing of hydrothermal $La_{0.5}Tb_{0.5}CrO_3$	15
S11 References	15

S1 Further Synthesis Details

The hydrothermal synthesis of $La_xSm_{1-x}CrO_3$ solid solutions is detailed elsewhere,[1] and the production of $La_{0.5}Tb_{0.5}CrO_3$ uses the same high-temperature hydrothermal treatment of an amorphous mixed-metal precursor. The amorphous precursor for $La_{0.5}Tb_{0.5}CrO_3$ was produced by dissolving the required molar ratios of lanthanum(III) nitrate hexahydrate (99.999%, Aldrich), terbium(III) nitrate hexahydrate (99.9%, Alfa Aesar), and chromium nitrate nonahydrate (99%, Aldrich) into deionised water. Addition of 2 M KOH solution caused the precipitation of a mixed-metal amorphous hydroxide gel, which was filtered and washed copiously with water, before being dried and ground into a fine powder. Approximately 350 mg of powdered precursor was then placed into an Inconel high-pressure vessel along with 20 ml of deionised water. The vessels were sealed and heated to 410 °C for 12 hours generating autogeneous pressures of 200+ bar, producing the bright green perovskite powders.

S2 Further Characterisation Details

Raman spectra were recorded in backscattering geometry using a Renishaw inVia Raman Microscope with spectral cutoff at ~120 cm^{-1} and equipped with a 632.8 nm He-Ne laser. A <1 mW laser power was used focused into a ~1 μm^2 spot to avoid local laser heating of the polycrystalline samples. Powders were placed in a Linkam THMS 600 sample stage and cooled to 123 K under liquid N_2.

Differential scanning calorimetry (DSC) was performed using platinum crucibles under a constant flow of N_2 (50 mL min^{-1}) on a Mettler Toledo Systems TGA/DSC1-1600 instrument. Data were recorded from room temperature up to 1400 °C at a rate of 20 °C min^{-1}.

S3 Crystallographic Data

Table S1. Crystallographic data for the $La_xTb_{1-x}CrO_3$ with x = 1, x = 0.5 and x = 0. Crystallographic data for $La_xSm_{1-x}CrO_3$ were reported previously.[1]

Crystallographic Data			
Formula Sum	$LaCrO_3$, *x* = 1.0	*Density, calculated*	6.758 g cm^{-3}
Formula Weight	238.90 g mol^{-1}	*Radiation*	Cu Kα_1, λ = 1.54056 Å
Crystal System	Orthorhombic	*Temperature*	298 K
Space Group	*Pnma* (no. 62)	R_p	8.287%
Z	4	R_{wp}	11.987%
Cell Volume	235.593(3) Å^3	*GOF*	1.158
Cell Dimensions	*a* = 5.48848(4) Å, *b* = 7.77796(6) Å, *c* = 5.51879(4) Å		

Atomic Coordinates

Atom	*Wyckoff*	*x*	*y*	*z*	U_{iso} / Å^2
La	4*c*	0.0188(2)	0.25	–0.0042(3)	0.0056(3)
Cr	4*b*	0	0	0.5	0.0015(4)
O (1)	4*c*	0.493(2)	0.25	0.064(2)	0.003(3)
O (2)	8*d*	0.273(2)	0.035(1)	0.725(2)	0.008(2)

Crystallographic Data			
Formula Sum	$La_{0.5}Tb_{0.5}CrO_3$, *x* = 0.5	*Density, calculated*	7.196 g cm^{-3}
Formula Weight	248.91 g mol^{-1}	*Radiation*	Cu Kα_1, λ = 1.54056 Å
Crystal System	Orthorhombic	*Temperature*	298 K
Space Group	*Pnma* (no. 62)	R_p	10.456%
Z	4	R_{wp}	13.320%
Cell Volume	229.74(3) Å^3	*GOF*	1.159
Cell Dimensions	*a* = 5.5097(2) Å, *b* = 7.6946(3) Å, *c* = 5.4190(2) Å		

Atomic Coordinates

Atom	*Wyckoff*	*x*	*y*	*z*	U_{iso} / Å^2
La/Tb	4*c*	0.0433(3)	0.25	–0.0107(9)	0.0057(9)
Cr	4*b*	0	0	0.5	0.0017(12)
O (1)	4*c*	0.485(3)	0.25	0.078(4)	0.0077(19)
O (2)	8*d*	0.289(4)	0.040(2)	0.714(5)	0.0007(19)

Crystallographic Data			
Formula Sum	$TbCrO_3$, *x* = 0.0	*Density, calculated*	7.756 g cm^{-3}
Formula Weight	258.92 g mol^{-1}	*Radiation*	Cu Kα_1, λ = 1.54056 Å
Crystal System	Orthorhombic	*Temperature*	298 K
Space Group	*Pnma* (no. 62)	R_p	7.448%
Z	4	R_{wp}	9.603%
Cell Volume	221.733(14) Å^3	*GOF*	1.105
Cell Dimensions	*a* = 5.52310(13) Å, *b* = 7.58067(15) Å, *c* = 5.29589(11) Å		

Atomic Coordinates

Atom	*Wyckoff*	*x*	*y*	*z*	U_{iso} / Å^2
Tb	4*c*	0.0605(3)	0.25	–0.0147(6)	0.0052(10)
Cr	4*b*	0	0	0.5	0.0037(14)
O (1)	4*c*	0.465(3)	0.25	0.096(3)	0.0050(15)
O (2)	8*d*	0.296(3)	0.0517(16)	0.699(3)	0.0019(15)

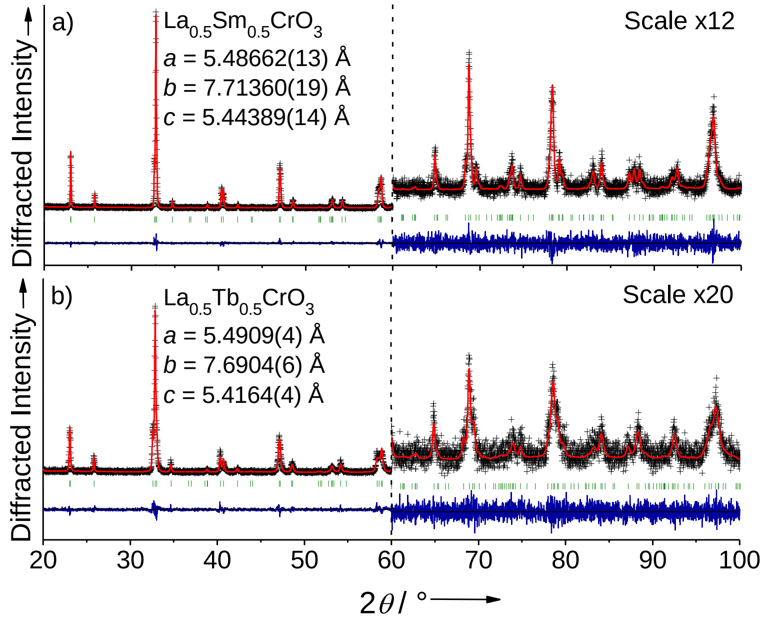

Figure S1. Rietveld refinements performed against PXRD data (λ = 1.54056 Å) of solid state samples of a) $La_{0.5}Sm_{0.5}CrO_3$ and b) $La_{0.5}Tb_{0.5}CrO_3$. The regions 60 ≤ 2θ ≤ 100 are scaled to display the fits at higher angle. Observed data (black crosses), calculated (red line), and difference (blue line) patterns are shown. Green tick marks denote positions of expected reflections for space group *Pnma*.

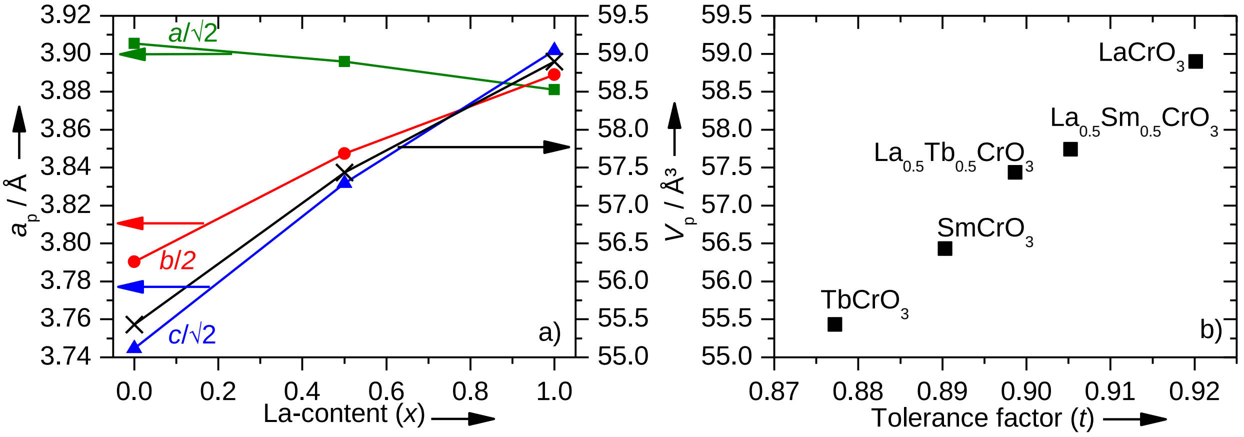

Figure S2. a) Variation of primitive unit cell parameters and volume with lanthanum content for $La_xTb_{1-x}CrO_3$ solid solutions synthesised hydrothermally. b) Plot of primitive cell volume against tolerance factor for all materials included in the study. Error bars are smaller than the data points.

Table S2. Lattice parameters of $La_xSm_{1-x}CrO_3$ and $La_xTb_{1-x}CrO_3$ x = 0.5 solid solutions produced through hydrothermal and solid state synthesis.

Material	Preparation	*a* / Å	*b* / Å	*c* / Å	*V* / Å³
$La_{0.5}Sm_{0.5}CrO_3$	Hydrothermal	5.49294(11)	7.71860(17)	5.44731(12)	230.954(9)
$La_{0.5}Sm_{0.5}CrO_3$	Solid State	5.48662(13)	7.71360(19)	5.44389(14)	230.394(9)
$La_{0.5}Tb_{0.5}CrO_3$	Hydrothermal	5.5097(2)	7.6946(3)	5.4190(2)	229.74(3)
$La_{0.5}Tb_{0.5}CrO_3$	Solid State	5.4909(4)	7.6904(6)	5.4164(4)	228.72(3)

S4 Raman Scattering

Raman scattering shows that for hydrothermal $La_{0.5}Tb_{0.5}CrO_3$, although measured at –150 °C to minimise thermal broadening, increased mode broadness is observed compared to both end members, arising from the variance disorder present as a result of La^{3+} and Tb^{3+} both occupying the A site. Despite the broadness, it is possible to discern the behaviour of some modes through the series. For example, the $B_{3g}(3)$ mode is associated with the stretching vibrations of the CrO_6 octahedra, and is therefore indirectly affected by structural distortion. The increase in Cr–O distances towards $TbCrO_3$ mean that the $B_{3g}(3)$ mode undergoes a softening with decreasing x. All other observed modes exhibit a greater dependence on structural distortion, and so soften towards the less-distorted $LaCrO_3$. The same mode broadening and dependence upon structural distortion were observed in the $La_xSm_{1-x}CrO_3$ solid solutions reported previously.[1]

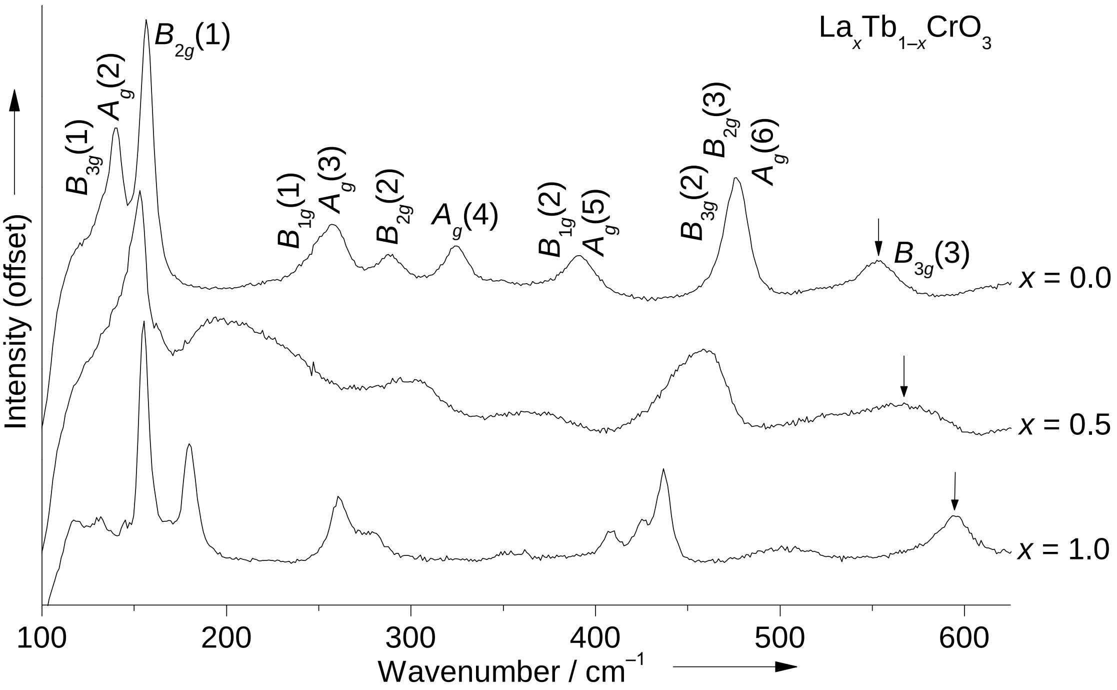

Figure S3. Raman spectra of $La_xTb_{1-x}CrO_3$ (x = 0, 0.5, and 1) recorded at –150 °C using a He-Ne laser line (632.8 nm). Mode assignments are for $TbCrO_3$, and arrows show the example progression of the $B_{3g}(3)$ mode through the series.

S5 Synthesis of $La_{0.5}Tb_{0.5}CrO_3$

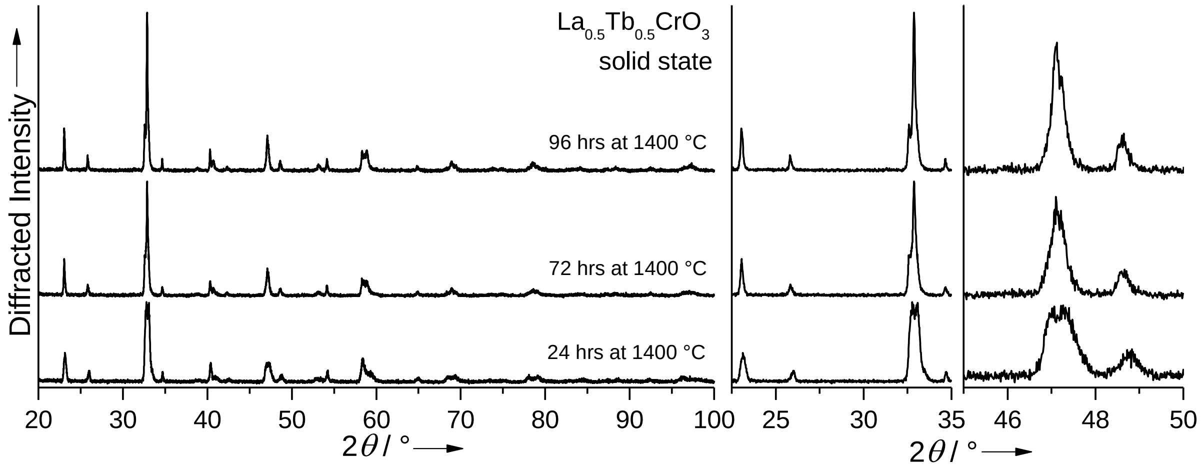

Figure S4. Ex situ PXRD patterns (λ = 1.54056 Å) of the solid state synthesis of $La_{0.5}Tb_{0.5}CrO_3$ showing that long firings at 1400 °C are required to produce a well reacted phase.

S6 Attempted syntheses of other mixed rare-earth chromites

Attempts at synthesising solid solutions with greater variance proved unsuccessful, as pure, crystalline samples could not be produced even at increased hydrothermal temperatures. The attempted synthesis of $La_{0.5}Ho_{0.5}CrO_3$ (σ^2 = 5.18×10^{-3} Å^2) produced a perovskite phase with broad reflections as well as a small amount of hydroxide impurity. It is possible that the reaction resulted in a mixture of lower A-site radius variance solid solutions, for example, an equimolar ratio of $La_{0.25}Ho_{0.75}CrO_3$ and $La_{0.75}Ho_{0.25}CrO_3$. However, this information is lost within the broad peaks.

An attempt at incorporating two lanthanides from opposite ends of the lanthanide series also proved impossible. $La_{0.5}Yb_{0.5}CrO_3$ (σ^2 = 7.57×10^{-3} Å^2) does not form, and instead a powder mixture of the end members $LaCrO_3$ and $YbCrO_3$ results. This suggests that the increased A-site radius variance of such systems led to these failed synthesis attempts.

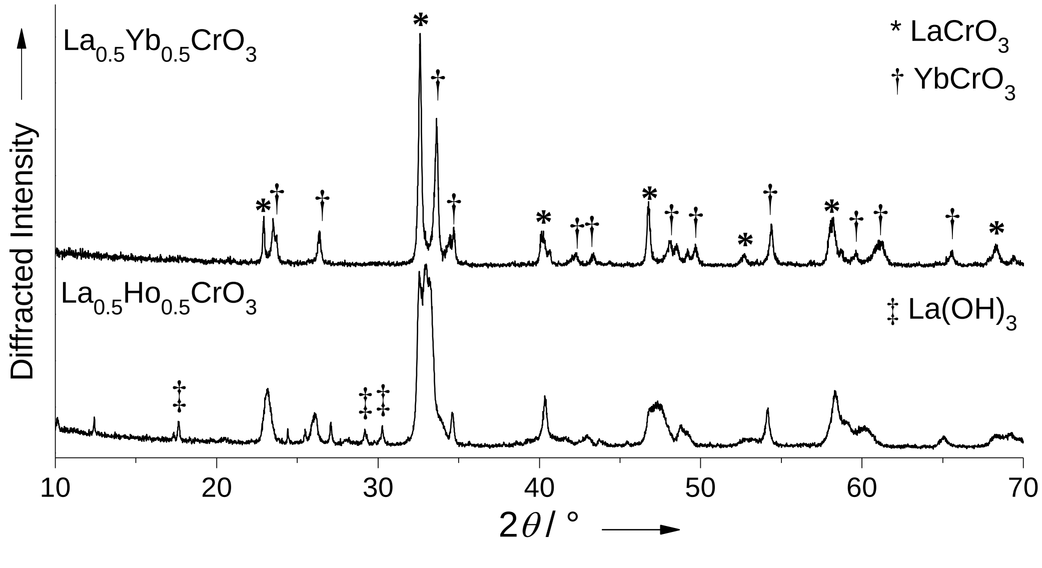

Figure S5. PXRD patterns (λ = 1.54056 Å) of phases produced from attempted hydrothermal syntheses of $La_{0.5}Yb_{0.5}CrO_3$ (upper) and $La_{0.5}Ho_{0.5}CrO_3$ (lower).

<u>**S7 HAADF-STEM & EELS**</u>

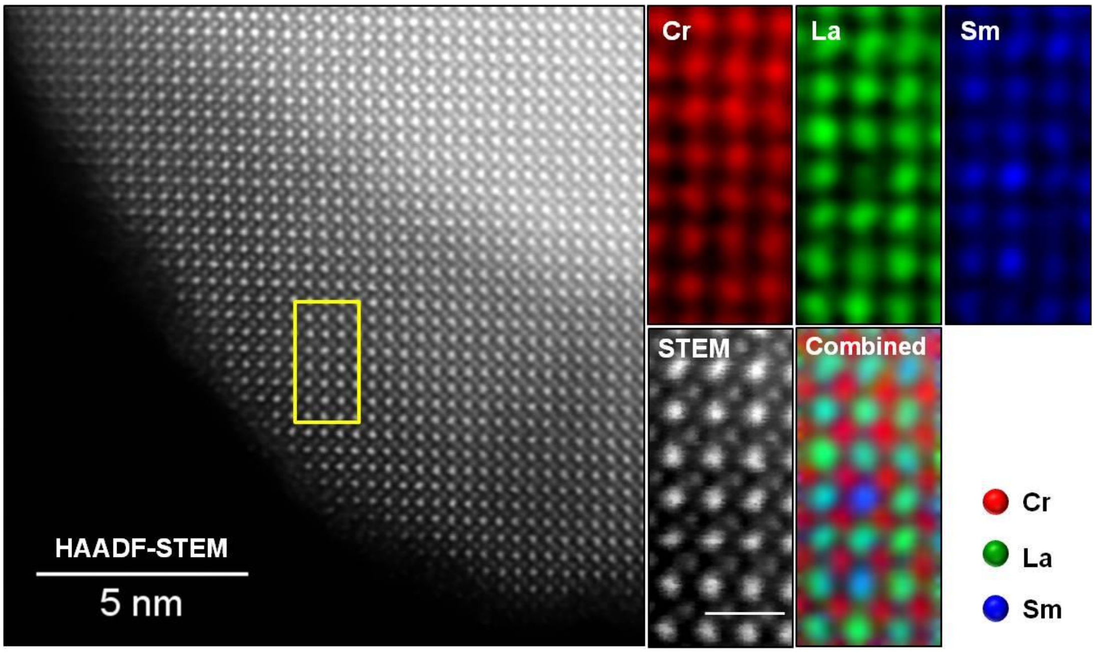

Figure S6. HAADF-STEM image (left) showing crystallite aligned along the [101] zone axis and the area selected for EELS (yellow box) on a thin section (approx. 40 nm) close to the crystal edge. On the right are separate integrated EELS maps for chromium (red), lanthanum (green), and samarium (blue), and all three combined into a single map. The white scale bar represents 1 nm.

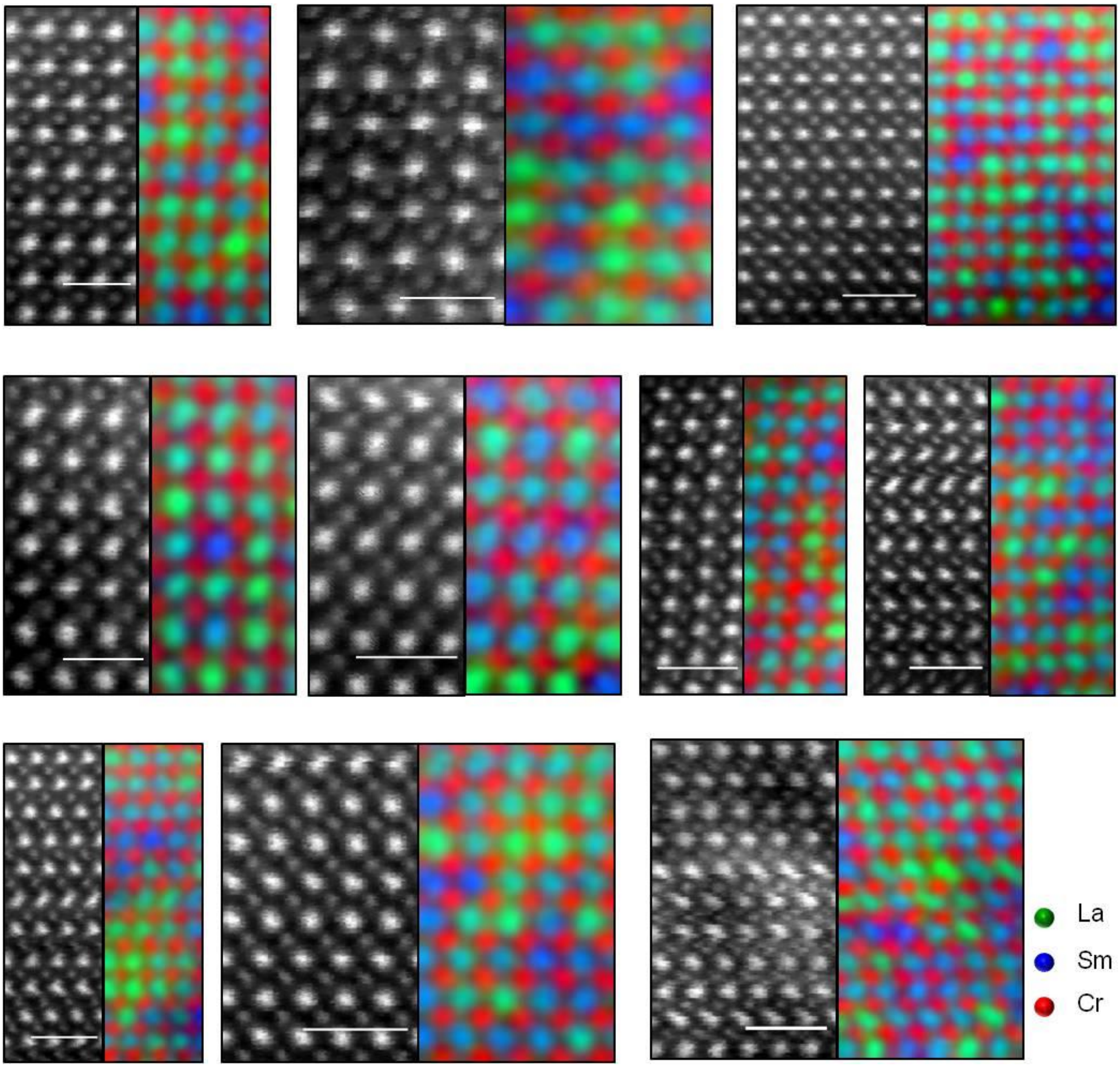

Figure S7. EELS maps formed from integrated La $M_{4,5}$, Sm $M_{4,5}$, and Cr $L_{2,3}$ edge spectra recorded from hydrothermal $La_{0.5}Sm_{0.5}CrO_3$ crystallites aligned along the [101] direction. Colours: lanthanum in green, samarium in blue, and chromium in red. White scale bars in each image represent 1 nm.

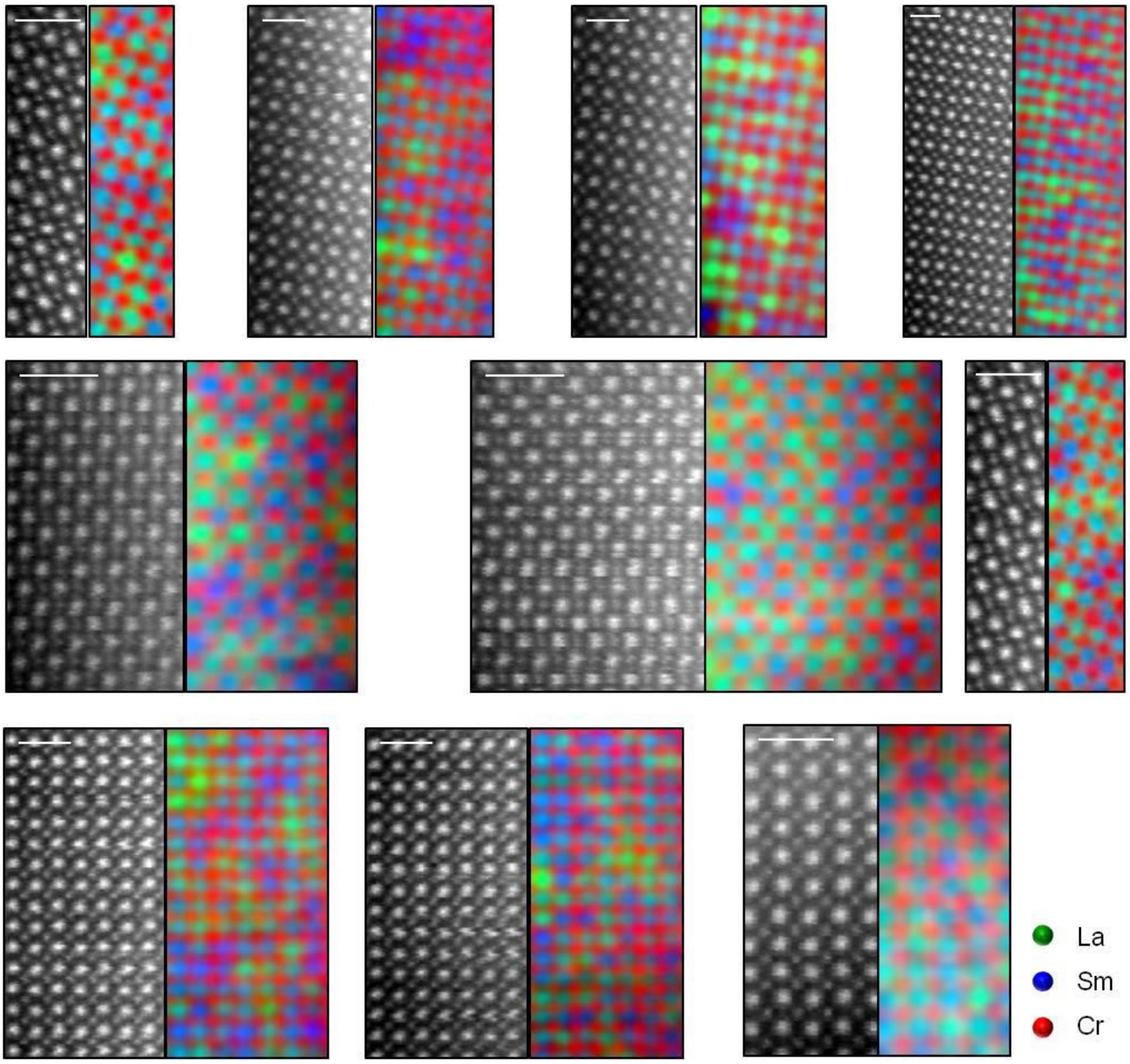

Figure S8. EELS maps formed from integrated La $M_{4,5}$, Sm $M_{4,5}$, and Cr $L_{2,3}$ edge spectra recorded from solid state $La_{0.5}Sm_{0.5}CrO_3$ crystallites aligned along the [101] direction. Colours: lanthanum in green, samarium in blue, and chromium in red. White scale bars in each image represent 1 nm.

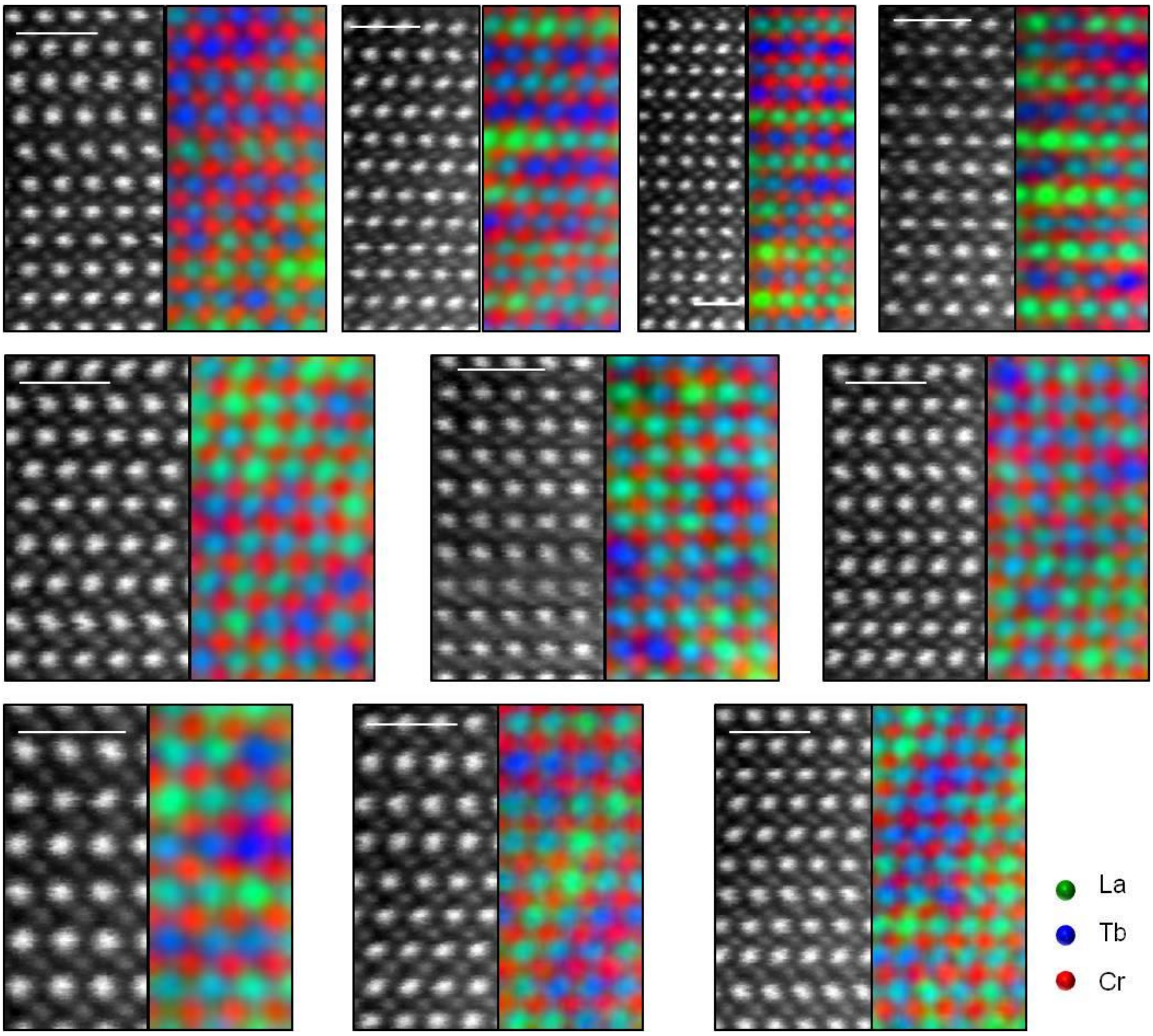

Figure S9. EELS maps formed from integrated La $M_{4,5}$, Tb $M_{4,5}$, and Cr $L_{2,3}$ edge spectra recorded from hydrothermal $La_{0.5}Tb_{0.5}CrO_3$ crystallites aligned along the [101] direction. Colours: lanthanum in green, terbium in blue, and chromium in red. White scale bars in each image represent 1 nm.

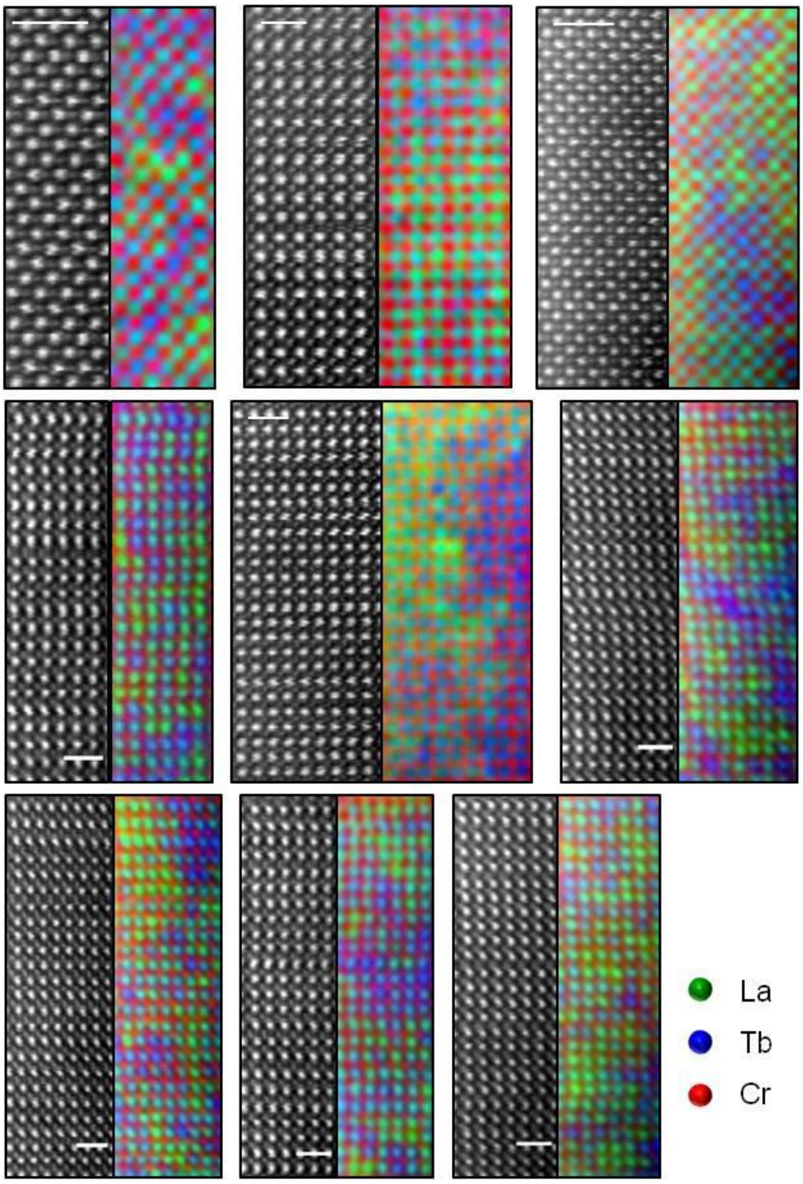

Figure S10. EELS maps formed from integrated La $M_{4,5}$, Tb $M_{4,5}$, and Cr $L_{2,3}$ edge spectra recorded from solid state $La_{0.5}Tb_{0.5}CrO_3$ crystallites aligned along the [101] direction. Colours: lanthanum in green, terbium in blue, and chromium in red. White scale bars in each image represent 1 nm.

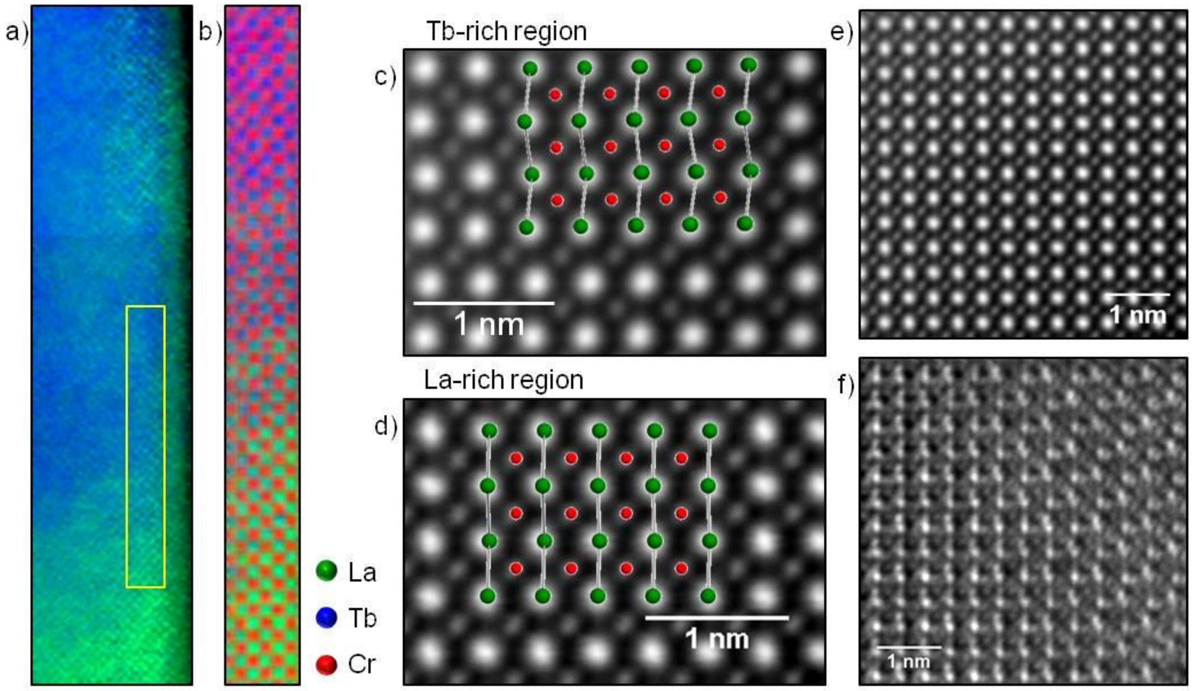

Figure S11. Large-scale a) and atomic-scale b) EELS maps of La-rich and Tb-rich domains observed in $La_{0.5}Tb_{0.5}CrO_3$ made from solid state synthesis. The map b) is a magnified section (yellow box) of a). HAADF-STEM images were recorded from both the Tb-rich c) and La-rich d) regions of this crystallite, and the structural overlays (lanthanide - green, chromium - red) show the increased octahedral distortion in c). Images e) and f) are HAADF and BF micrographs, respectively, across the boundary between the two domains. BF imaging is more sensitive to electron scatter from light elements (i.e. oxygen) and so the boundary is more apparent in f) as the structural distortions between $LaCrO_3$ and $TbCrO_3$ manifest themselves mostly through anion positions.

S8 Differential Scanning Calorimetry (DSC)

Differential scanning calorimetry (DSC) was performed on the $La_{0.5}Tb_{0.5}CrO_3$ materials following different synthetic treatments in order to investigate the possiblity of thermal randomisation of the A site cation order observed in hydrothermal $La_{0.5}Tb_{0.5}CrO_3$. The DSC traces over the temperature range 1050 - 1350 °C show clear differences with an exothermic destabilisation of the local A site layers observed in the hydrothermal sample at approximately 1200 °C. This supports the magnetic susceptibility measurements discussed in the main text, where changes are observed between hydrothermal and solid state $La_{0.5}Tb_{0.5}CrO_3$, and with each subsequent annealing.

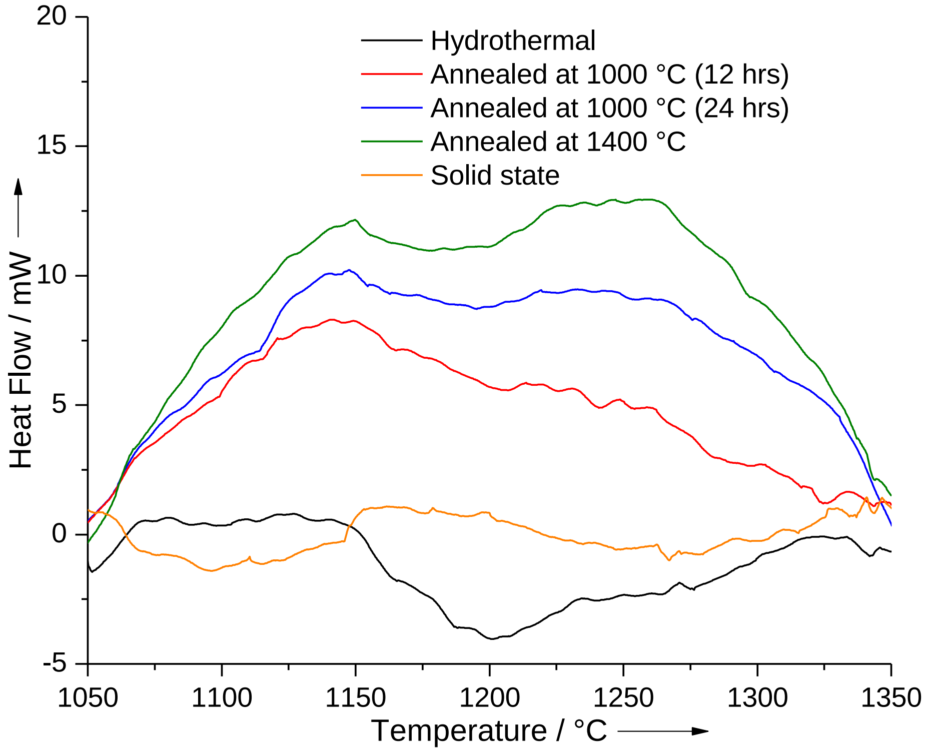

Figure S12. Comparison between DSC traces of as-made hydrothermal $La_{0.5}Tb_{0.5}CrO_3$ and subsequent annealed materials.

S9 Magnetic susceptibility

Field-cooled cooling (FCC) magnetic susceptibility data were recorded on materials in the $La_xTb_{1-x}CrO_3$ series prepared hydrothermally. FCC data for the $La_xSm_{1-x}CrO_3$ were reported previously.[1]

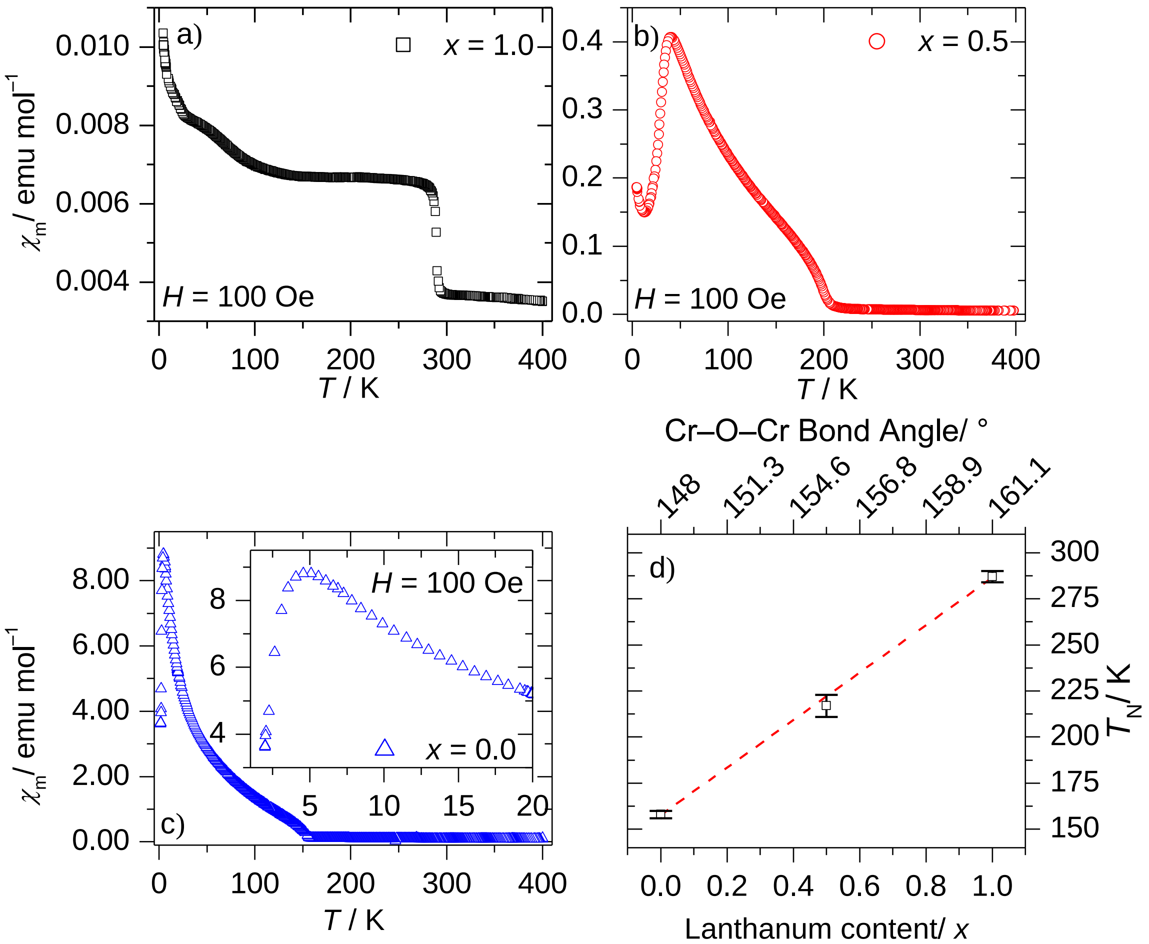

Figure S13. FCC curves for hydrothermal $La_xTb_{1-x}CrO_3$ materials, measured in applied fields of 100 Oe. Figures a), b), and c) show data for x = 1.0, 0.5, and 0.0, respectively. The inset in c) highlights the low temperature ordering of Tb^{3+} moments at ~5 K. Panel d) shows the variation of T_N as a function of composition and superexchange bond angle.

S10 Annealing of hydrothermal $La_{0.5}Tb_{0.5}CrO_3$

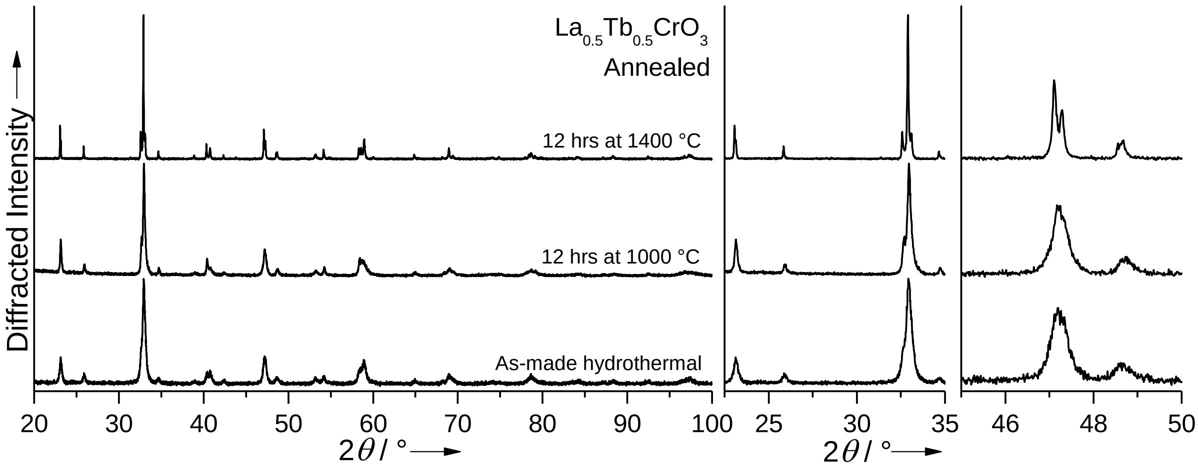

Figure S14. PXRD patterns (λ = 1.54056 Å) of annealed hydrothermal $La_{0.5}Tb_{0.5}CrO_3$ material. The final firing at 1400 °C results in more resolved peaks than that of conventional ceramic synthesis.

S11 References

[1] L. M. Daniels, M. C. Weber, M. R. Lees, M. Guennou, R. J. Kashtiban, J. Sloan, J. Kreisel, R. I. Walton, *Inorg. Chem.* **2013**, *52*, 12161-12169.
